# Phytochrome B-mediated activation of lipoxygenase modulates an excess red light-induced defence response in *Arabidopsis*

**DOI:** 10.1093/jxb/eru247

**Published:** 2014-06-10

**Authors:** Yuanyuan Zhao, Jun Zhou, Da Xing

**Affiliations:** MOE Key Laboratory of Laser Life Science & Institute of Laser Life Science, College of Biophotonics, South China Normal University, Guangzhou 510631, PR China

**Keywords:** *Arabidopsis*, defence response, excess red light, lipoxygenase, MAPK, phytochrome B.

## Abstract

Lipoxygenase (LOX), a non-haem-iron-containing dioxygenase, is activated under various biotic or abiotic stresses to trigger a series resistance response, but the molecular mechanism of LOX activation remains unclear. This work investigated the activation of LOX during the plant defence response induced by excess red light (RL). In conditions of RL-induced defence, *Arabidopsis* LOX activity and transcription levels of *LOX2*, *LOX3*, and *LOX4* were both upregulated. Under RL, phytochrome B promoted the degradation of phytochrome-interacting factor 3 (PIF3), a factor that inhibited the expression levels of *LOX*s, and thus the transcription levels of *LOX2*, *LOX3*, and *LOX4* were increased. Upon pathogen infection, the activity of mitogen-activated protein kinase 3 (MPK3) and MPK6 was increased in plants pre-treated with RL. Moreover, experiments with the inhibitor PD98059 and mutants *mpk3* and *mpk6-2* demonstrated that MPK3 and MPK6 were both responsible for LOX activation. Further results showed that, in response to RL, an increase in cytoplasmic calcium concentration and upregulation of *calmodulin 3* (*CaM3*) transcript level occurred upstream of MPK3 and MPK6 activation. Collectively, these results suggested that activation of LOX both at the transcript level and in terms of activity modulates the defence response induced by RL, providing a new insight into the mechanistic study of LOX during plant defences.

## Summary

Excess red light induces a defence response by activating lipoxygenase at both the transcript and activity levels.

## Introduction

Plants, as sedentary organisms, have evolved a high flexibility in both metabolism and development to cope with the multiple environmental stimuli that they are exposed to (Genoud *et al.*, 2002). Light signalling is fundamental to the growth and development of plants. Long-term exposure to either low light or excess white light (EL) has an adverse effect on plants. However, certain irradiation with moderate EL can enhance the plant defence response, and red light (RL) plays a major role (Szechyńska-Hebda *et al.*, 2010). Phytochrome B (phyB), as the main receptor of RL, is essential for this process. Szechyńska-Hebda et al. (2010) found that photo-electrophysiological signalling is a component of signalling cascades that potentially regulates the defence response. However, the mechanisms mediating the defence response by phyB are still unclear. Plants monitor informational light signals from their surroundings using a range of sensory photoreceptors including phototrophin, crytochrome, and phytochrome (Leivar *et al.*, 2012). RL and far-RL are sensed using the phytochrome family (phyA to phyE in *Arabidopsis*). Phytochromes perceive RL (660nm) and far-RL (720nm) of the solar spectrum, and monitor changes in light quality and quantity to control many aspects of growth and developmental responses such as germination, seedling de-etiolation, shade avoidance, and flowering time (Franklin and Quail, 2010; Strasser *et al.*, 2010). Phytochromes photoconvert between two conformers reversibly: the inactive RL-absorbing Pr form and the biologically active far-RL-absorbing Pfr form. Photoconversion of Pr to Pfr takes place upon absorption of RL (Linschitz *et al.*, 1966), and reversion of Pfr to Pr occurs in far-RL-enriched circumstances.

The Pr form of phytochromes is synthesized in the cytoplasm, and upon photoactivation to Pfr is translocated to the nucleus (Nagatani, 2004), where it associates with phytochrome-interacting factors (PIFs) (Soy *et al.*, 2012). PIFs, a subset of basic helix–loop–helix transcription factors, preferentially bind with a G-box (CACGTG) DNA sequence element, which is a subclass of an E-box element (CANNTG) present in the promoters of some light-regulated genes. Interactions between the Pfr form of phyB and PIF3 bound to a G-box promoter motif are hypothesized to directly regulate transcript expression of light-responsive genes (Martínez-García *et al.*, 2000; Quail, 2002).

Lipoxygenases (LOXs) catalyse peroxidation of many polyunsaturated fatty acids and some lipids to cause the production of oxylipins, a set of biologically active compounds (Yang *et al.*, 2012). Oxylipins have many important physiological functions during signalling transduction in growth and development, senescence and death, and biotic or abiotic stress responses (Feussner and Wasternack, 2002; Porta and Rocha-Sosa, 2002; Duan *et al.*, 2005; Liavonchanka and Feussner, 2006). The many different products of LOX could enhance the defence responses in plants, including direct inhibition of the pathogen and accumulation of phytoalexins (Alami et al, 1999; Lin and Ishii, 2009). There are six isoforms of LOX in *Arabidopsis*, and these can be classified as 9-LOXs or 13-LOXs according to the position at which oxygen is incorporated into substrates for LOX catalysis in plants (Feussner and Wasternack, 2002). LOX1 and LOX5 are 9-LOXs, while LOX2, LOX3, LOX4, and LOX6 are 13-LOXs (Bannenberg *et al.*, 2009). As a key enzyme in the lipid peroxidation reaction, LOX plays an important role during the defence response. Its expression level dramatic rises in response to EL, indicating that it may play a role in this process (Rossel *et al.*, 2007). However, whether LOX’s upregulation of transcript levels is induced by a specific spectrum or by EL in general is still unknown.

The *LOX* gene sequence may contain a G-box or a similar domain structure (Hou *et al.*, 2010), which is assumed to combined with PIFs. This assumption provides a possible mechanism underlying the regulation of *LOX* gene expression by excess RL, i.e. RL promotes the degradation of PIFs, which suppress *LOX* transcription by combining with it, and the inhibited *LOX* is released, thus contributing to the increase in *LOX* transcript expression.

Protein kinases and phosphatases play a central role in signal transduction through the phosphorylation and dephosphorylation of proteins. The mitogen-activated protein kinase (MAPK) cascade, as the most conversed pathway, plays a crucial role in almost all eukaryotes by linking perception of external stimuli with changes in the cell (Taj *et al.*, 2010). Each MAPK cascade consists of at least three kinases: MAPKKK, MAPKK, and MAPK. In the *Arabidopsis* genome, there are 20 MAPKs, 10 MAPKKs, and ~80 MAPKKKs (Colcombet and Hirt, 2008; Beckers *et al.*, 2009). They play a pivotal role in the transduction of various extracellular stimuli, including many biotic and abiotic stresses, as well as a series of developmental responses (Taj *et al.*, 2011b). Many studies in the literature have demonstrated that MAPKs (MPKs) take part in the regulation of innate immunity and adverse stress responses (Ichimura *et al.*, 2002; Xing *et al.*, 2008). Activation of MPK has been detected under different stimuli, for example, MPK3 and MPK6 are activated under some abiotic stresses, such as ethylene treatment, drought, or wounding; MPK6 is activated under heat shock; MPK4 and MPK6 are activated under conditions of cold, salt, or H_2_O_2_; and when under heavy metal stress, MPK2, MPK3, MPK4, and MPK6 are all activated. Infection with different kinds of pathogens can induce different pathways of MPKs. For example, fungal pathogens can induce MPK2, MPK3, MPK4, and MPK6 and bacterial pathogens can induce MPK2, MPK3, and MPK6 under normal circumstances (Taj *et al.*, 2010). It is still unclear whether the most active MPK3 and MPK6 are activated during the enhancement of the defence response induced by RL. Ca^2+^ is a crucial second messenger (Hashimoto and Kudla, 2011), and previous studies have implicated it in the activation of MPK cascades during the response to various stimuli (Xing *et al.*, 2008; Wang PC et al., 2010). The Ca^2+^ signatures are sensed, decoded, and transmitted to downstream signalling cascades by Ca^2+^ sensors. Calmodulin (CaM) acts as a prominent Ca^2+^ sensor protein in plant signal transduction. In *Arabidopsis*, CaM has several isoforms, and different isoforms interact with their particular targets upon different exogenous stimuli (Luan *et al.*, 2002). The Ca^2+^ signature is believed to be necessary for the cellular signalling transduction in response to EL. However, analysis of the Ca^2+^–CaM response to RL, especially the signalling pathway leading to the defence reaction, is still lacking.

In this study, the possible molecular mechanisms underlying the process of RL-induced activation of LOX during enhancement of the defence response were investigated. Our results indicated that LOX was upregulated by RL at both the transcriptional level and activity level, and that it plays an important role in the RL-induced defence response.

## Materials and methods

### Plant materials and chemicals

*Arabidopsis* ecotype Columbia-0 (Col-0) and seeds of mutants *phyB*, *mpk3*, and *mpk6-2* were obtained from the European *Arabidopsis* Stock Centre. *phyB-ox-YFP* (Wang FF et al., 2010), *pif3* and *pif3-ox-YFP* (Soy *et al.*, 2012) were sterilized and grown on solid Murashige and Skoog medium as described previously (Zhang and Xing, 2008). 4,′(2, 3-Dimethyltetramethylene)dipyrocatechol (NDGA) was obtained from Merk, linoleic acid, 1,2-bis(2-aminophenoxy)ethane-*N*,*N*,*N*′,*N*′-tetra-acetic acid (acetoxymethyl ester) (BAPTA-AM), and PD98059 were purchased from Sigma-Aldrich. Fluo-3-AM was obtained from Beyotime.

### RL treatment

*Arabidopsis* rosettes were fully exposed to EL (1500 μmol photons m^–2^ s^–1^ for 1h) and excess RL (120 μmol photons m^–2^ s^–1^ for 4h, 650±20nm) supplied by light-emitting diode panels (Photon System Inst.). The above light conditions provided similar energy at the indicated spectral regions. Heat emission from the light source was insignificant.

### Pathogen growth and inoculation

The bacterial strain used in this study was *Pseudomonas syringae* pv. *tomato* DC3000 (Pst-DC3000, virulent), and it was grown at 28 °C in King’s B medium supplemented with appropriate antibiotics. Overnight log-phase cultures were collected by centrifugation, washed with 10mM MgCl_2_, and then diluted to a final optical density at 600nm (OD_600_) of 0.01 (for appearance determination) and 0.0001 (for pathogen growth assay). The procedures of pathogen inoculation and bacteria growth assays were as described previously (Mishina and Zeier, 2007).

### Callose staining

The leaves of approximately 4-week-old plants of wild-type (WT), *phyB*, or *phyB*-*ox*-*YFP* were fixed in ethanol:acetic acid (3:1, v/v) and stained with 0.01% (w/v) aniline blue (Millet *et al.*, 2010). The leaves were mounted on slides, and callose was observed with UV excitation (Sun *et al.*, 2012).

### RNA extraction and reverse transcription (RT)-PCR analysis

Total RNAs were extracted from detached *Arabidopsis* leaves using Trizol according to the supplier’s recommendations. The concentration of RNA was determined by measuring absorbance at 260nm. First-strand cDNA was synthesized with a SuperScript II First-Strand Synthesis System for qRT-PCR (Invitrogen). Quantitative RT-PCR was performed using a Roche LightCycler^TM^ 2.0 Real-time Detection System. The expression of target gene was normalized relative to the housekeeping gene *ACTIN2* (Zhou *et al.*, 2013). The primers used are listed in Supplementary Table S1 at *JXB* online.

### LOX activity assays

The assay of LOX activity was performed as described previously (Skórzyńska-Polit *et al.*, 2006) with a minor modification. The plant material was deep frozen in liquid nitrogen and ground in 0.2M boric acid buffer at pH 7.0. The homogenate was centrifuged at 15 000*g* for 20min, and the supernatant was used for determination of protein concentration and LOX activity. Protein concentration was determined according to Bradford (1976) using a Bio-Rad protein assay using BSA as a standard. The activity of LOX was measured spectrophotometrically at 234nm as described previously (Skórzyńska-Polit and Krupa, 2003). The reaction mixture contained 0.2M boric acid buffer (pH 8.0), 25 μl of plant extract, and 25 μl of linoleic acid as a substrate in a final volume of 1ml. The reaction was carried out at 30 °C for 4min. LOX activity was expressed as absorbance increase mg^–1^ of protein min^–1^.

### Treatment with LOX inhibitor and MAPK cascade inhibitor

Before RL treatment and the later inoculation of Pst-DC3000, the *Arabidopsis* leaves were pre-sprayed with a solution containing NDGA (an inhibitor of LOX; dissolved in DMSO) or PD98059 (an inhibitor of the MAPK pathway; dissolved in DMSO) for 60min (Samuel *et al.*, 2000; Matsumura *et al.*, 2003; Miles *et al.*, 2004). NDGA was used at a final concentration of 100 μM to inhibit the activity of LOX. A concentration gradient experiment was carried out for inhibitor PD98059 (Fig. S3). The results indicated that a concentration of 20 µM PD98059 could markedly inhibit the induction of LOX activity by inhibiting kinase activity, so this concentration was used in subsequent experiments.

### Western blot and MAPK activity assay

Proteins were extracted from detached *Arabidopsis* leaves at the indicated time points after different treatments. Protein extracts were separated on a 10% SDS-PAGE mini-gel and then analysed by Western blotting. For detection of the phosphorylated proportion of MAPKs, as described by Li *et al.* (2012), blots were probed with anti-ACTIVE MAP kinase polyclonal Ab (pTEpY; Cell Signaling Technology, MA, USA), which recognizes activated MAPKs. Western blot experiments were carried out to show the MPK3/6 activity of the *mpk6-2* and *mpk3* mutant lines (Fig. S4). The results showed that the *mpk6-2* mutant did not have MPK6 activity (47kDa) and *mpk3* mutants did not have MPK3 (43kDa) activity compared with the WT. Together, these results demonstrated that the two bands were MPK3 and MPK6, so we used this antibody in our experiments. Subsequently, the blots were washed and incubated with an anti-rabbit horseradish peroxidase-conjugated secondary antibody.

### Measurement of cytosolic calcium concentration ([Ca^2+^]_cyt_)

The method for [Ca^2+^]_cyt_ detection was based on previous work (Zhou *et al.*, 2013). Ca^2+^ was stained with Fluo-3-AM, which is hydrolysed to yield Fluo-3 capable of indicating changes in [Ca^2+^]_cyt_. The fluorescence intensity of Fluo-3 was measured by flow cytometry analysis.

### Chromatin immunoprecipitation (ChIP)

For ChIP analysis, samples (1.5g) were cross-linked with 10ml of 1% formaldehyde under vacuum infiltration conditions. ChIP assays were performed as described previously (Shin et al, 2007) with a minor modifications of the three wash buffers: low wash buffer (150mM NaCl, 0.1% SDS, 1% Triton X-100, 2mM EDTA, 20mM Tris/HCl, pH 8), high-salt wash buffer (500mM NaCl, 0.1% SDS, 1% Triton X-100, 2mM EDTA, 20mM Tris/HCl, pH 8), and LiCl wash buffer (0.25M LiCl, 1% v/v NP-40, 1% w/v sodium deoxycholate, 1mM EDTA, 10mM Tris/HCl, pH 8). The amount of each precipitated DNA fragment was determined by semi-quantitative PCR using *LOX2*, *LOX3*, and *LOX4* primers.

### Co-immunoprecipitation assay

A co-immunoprecipitation assay was performed as described previously (Liu *et al.*, 2013) with some modifications. Total proteins were extracted from plants in extraction buffer [50mM Tris/HCl, pH 7.5–8.0, 100mM NaCl, 1% NP-40, 0.5% sodium deoxycholate, 0.1% SDS, 1mM EDTA, 1mM sodium orthovanadate, 50mM sodium fluoride, and 1mM phenylmethylsulfonyl fluoride, containing Protease Inhibitor Cocktail (Roche)]. Proteins extracts were inoculated with antibody for 4h, and protein A beads were then added. After incubation overnight at 4 °C, the beads were centrifuged and washed four times with PBS (pH 7.4). The immunoprecipitated proteins were detected by SDS-PAGE with an anti-ERK (extracellular signal-regulated kinase) antibody. Endogenous LOX protein was detected with a rabbit polyclonal anti-LOX antibody (Stenzel *et al.*, 2003) after immunoprecipitation with anti-ERK antibody.

### GenBank accession numbers

Sequence data from this article can be found in GenBank under the following accession numbers: *PR1*, AT2g14610; *calmodulin 3* (*CaM3)*, AT3g56800; *Actin2*, AT3g46520; *LOX1*, AT1g55020; *LOX2*, AT3g45140; *LOX3*, AT1g17420; *LOX4*, AT1g72520; *LOX5*, AT3g22400; *LOX6*, AT1g67560.

## Results

### EL protects *Arabidopsis* from pathogen infection, while RL is the main inducer

**Fig. 1.**
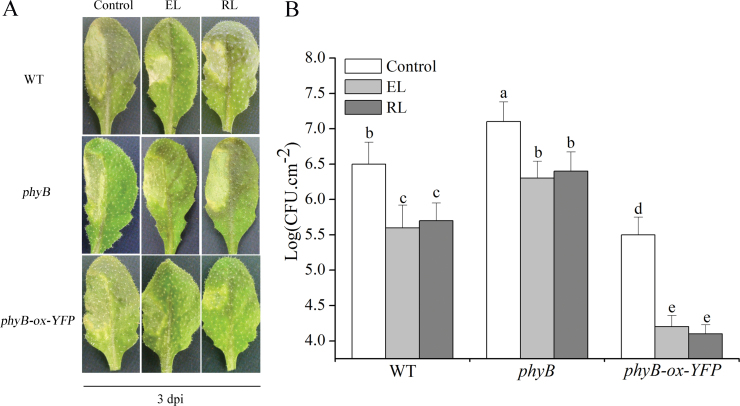
Effect of exposure to EL and RL on disease progression in leaves of WT, *phyB*, and *phyB-ox-YFP* plants. (A) After exposure to EL (1500 μmol photons m^–2^ s^–1^for 1h), and excess RL (120 μmol photons m^–2^ s^–1^ for 4h), WT and *phyB*, *phyB-ox-YFP* plants were inoculated with virulent Pst-DC3000 (OD_600_=0.01 in 10mM MgCl_2_). Leaves were infected on their left halves, and samples were collected at 3 d post-inoculation (dpi). (B) Bacterial growth quantification of Pst*-*DC3000-inoculated (OD_600_=0.0001) leaves after exposure to EL and RL. Samples were collected at 3 dpi for the assay. Each value is the mean±standard deviation (SD) of three replicates. Different letters indicate statistically significant differences between treatments (Duncan’s multiple range test: *P*<0.05). CFU, colony-forming units. (This figure is available in colour at *JXB* online.)

It has been demonstrated that EL regulates plant stress responses. The results of Szechyńska-Hebda et al. (2010) indicated that red but not blue EL induces a defence response. When *Arabidopsis* plants were inoculated with virulent Pst-DC3000, the WT plant leaves under normal light conditions turned yellow and finally wilted and died (Fig. 1A), whereas plants pre-irradiated with EL (1500 μmol m^–2^ s^–1^ for 1h) or excess RL (120 μmol m^–2^ s^–1^ for 4h; 660–680nm) showed minute yellow disease lesions at 3 d post-inoculation (dpi). The *phyB* null mutant plants showed more developed chlorotic lesions compared with WT plants, and RL-treated *phyB* plants showed no significant improvement on disease progression at 3 dpi. However, the chlorotic lesions were reduced in *phyB-ox-YFP* plants, which are transgenic plants overexpressing phyB::YFP (yellow fluorescent protein) fusion protein, and both EL and RL made this process more significant. In addition, the numbers of bacteria were significantly reduced in WT plants pre-irradiated with both EL and RL but not in *phyB* plants, compared with WT, and the reduction was more significant in *phyB-ox-YFP* plants (Fig. 1B). This finding was consistent with the disease symptoms shown in Fig. 1A. For the pathogen growth assays in Fig. 1B, the *phyB Arabidopsis* plants were more susceptible than WT plants, and either EL or RL had nearly the same impact on limitation of bacterial numbers. Together, these results illustrated that EL can induce an *Arabidopsis* defence response to pathogen and that excess RL is the main inducer, while phyB plays an important role during induction of the defence response.

### RL-induced defence responses are dependent on LOX activity

**Fig. 2.**
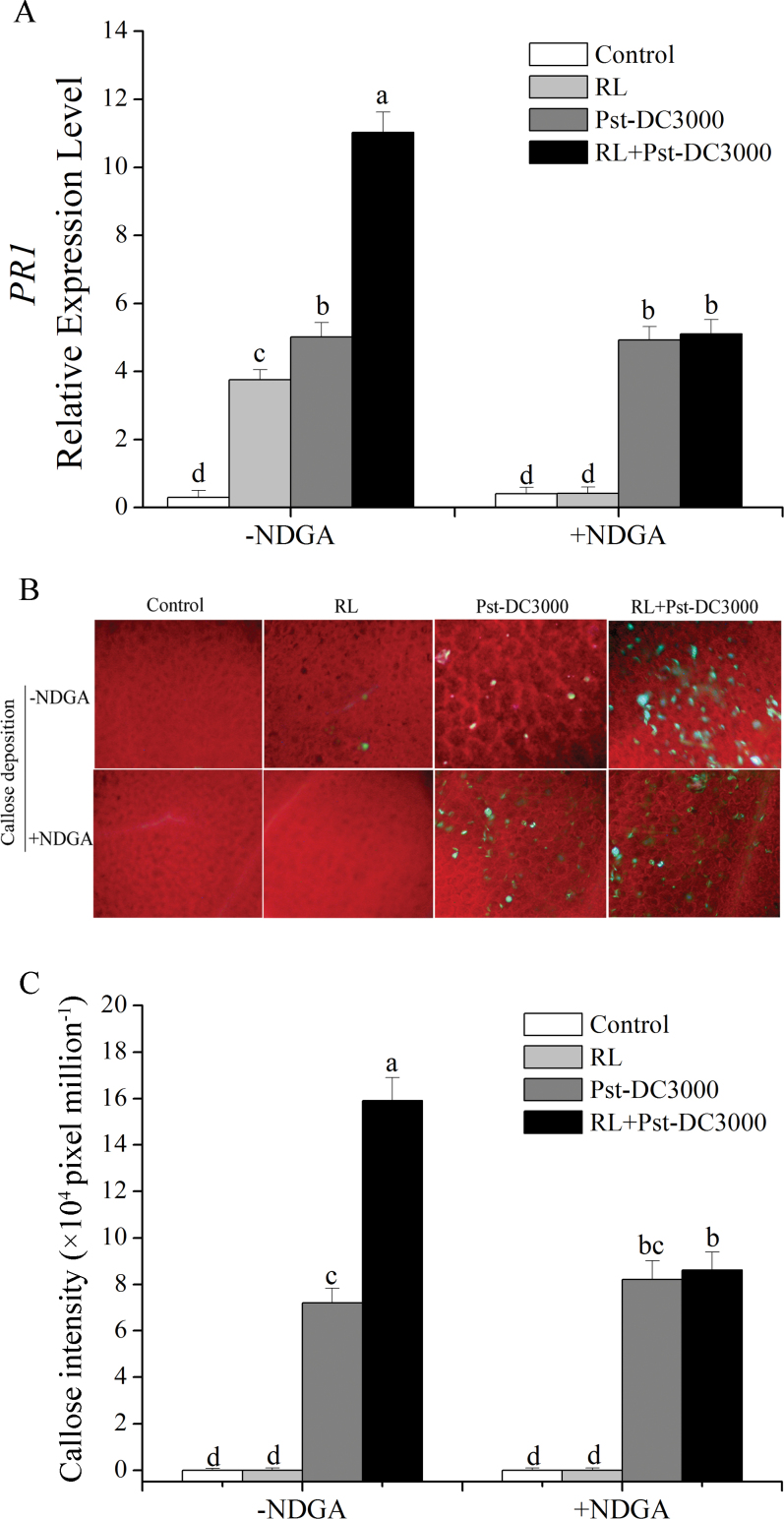
Effect of LOX inhibitor (NDGA) on the RL-induced defence response in *Arabidopsis*. (A) Quantitative RT-PCR data showing the influence of suppression of LOX activity on *PR1* gene expression during the defence response in WT plants. Plants were pre-sprayed with or without NDGA (50 μM) and treated as follows: control, no treatment; RL, 120 μmol photons m^–2^ s^–1^ for 4h; Pst-DC3000 inoculation, OD_600_=0.01 in 10mM MgCl_2_; RL+Pst-DC3000, inoculation after RL. (B) Callose-staining imaging of leaves from plants under different treatments. (C) Callose deposition in the leaves in (B) was quantified by determining the number of pixels per million pixels in digital photographs. Data are means±SD of three experiments. Different letters indicate statistically significant differences between treatments (Duncan’s multiple range test: *P*<0.05). (This figure is available in colour at *JXB* online.)

In our research, as the markers of enhancement of defence response, transcript expression of *PR1* (pathogenesis-related protein 1) and deposition of callose were analysed. First, we detected the effect of different pre-treatment times of RL on plant resistance to pathogen infection, with transcript expression levels of *PR1* as the indicator (Supplementary Fig. S1 at *JXB* online). We found that the expression level increased significantly with the extension of RL treatment time and the level gradually became stable, which indicated that 4h of RL could activate the plant defence response against the invading pathogen. Therefore, we chose RL (120 μmol m^–2^ s^–1^ for 4h; 660–680nm) as the inducer of the defence response in the following experiments. As shown in Fig. 2A, analysis of *PR1* gene expression by RT-PCR revealed that, when plants were inoculated with Pst-DC3000, the *PR1* transcript was significantly increased in plants with pre-irradiation of RL, whereas a lower level of expression was found in plants without RL. However, when plants were pre-treated with NDGA, a non-selective inhibitor of LOX (Xu *et al.*, 2005; Keereetaweep *et al.*, 2010; Gao *et al.*, 2011; Wang *et al.*, 2012), the induction of *PR1* transcripts was insignificant. We also detected callose deposition. When plants were inoculated with Pst-DC3000, more callose deposition was observed in plants pre-treated with RL than in plants without exposure to RL. Pre-treatment with NDGA could erase the deposition of more callose induced by RL (Fig. 2B, C). The results in Fig. 2 indicated that NDGA application alone did not induce any evident responses in plants, whereas RL-induced defence responses were arrested by the inhibitor. Clearly, RL treatment primes *Arabidopsis* plants for augmented induction of defence responses when challenged by the pathogen, and activation of LOX was necessary during the process.

### Expression and activity of LOX are induced by RL

**Fig. 3.**
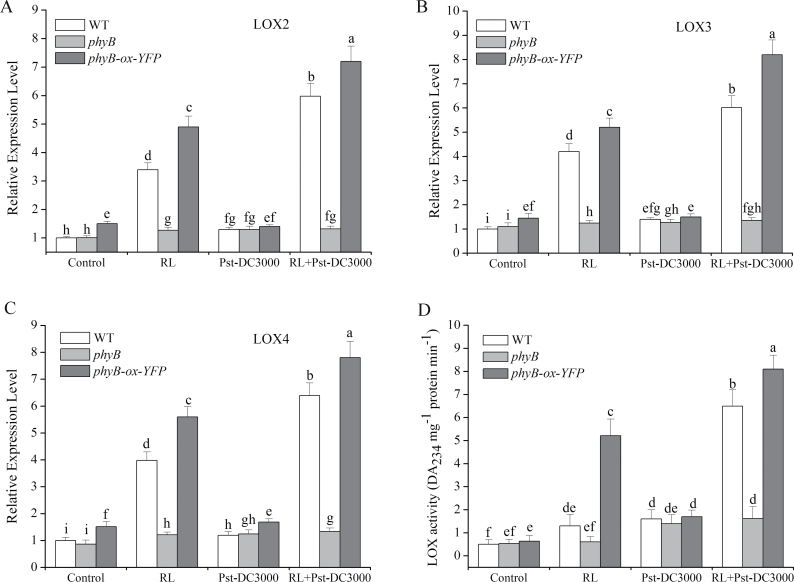
Induction of transcription levels and activity of LOX by RL. (A–C) Transcript levels of *LOX2*, *LOX3*, and *LOX4* in WT, *phyB*, and *phyB-ox-YFP* plants. (D) LOX activity analysis in WT, *phyB*, and *phyB-ox*-*YFP* plants. Total RNA and proteins were extracted from the leaves of full-grown *Arabidopsis* after different treatments as follows: control, no treatment; RL, 120 μmol photons m^–2^ s^–1^ for 4h; Pst-DC3000 inoculation, OD_600_=0.01 in 10mM MgCl_2_; RL+Pst-DC3000, inoculation after RL. *Arabidopsis ACTIN2* was used as an internal control. Different letters indicate statistically significant differences between treatments (Duncan’s multiple range test: *P*<0.05). Values represent means±SD of three independent experiments.

Application of the LOX inhibitor NDGA had a negative influence on the enhancement of defence induced by RL (Fig. 2). This was preliminary evidence that the induction of defence was dependent on LOX. To verify this further, we detected expression of six isoforms of *LOX* in *Arabidopsis* (Fig. 3A–C and Supplementary Fig. S2 at *JXB* online). The elevated expression levels of *LOX2*, *LOX3*, *LOX4* were most evident in plants with RL treatment before Pst-DC3000 inoculation in comparison with either RL treatment only or Pst-DC3000 inoculated directly (Fig. 3A–C). Conversely, such a reinforcing effect was ruled out in *phyB* plants and amplified in *phyB-ox-YFP* plants. Transcripts of *LOX1*, *LOX5*, and *LOX6* were almost unaffected by RL (Fig. S2). Examination of LOX activity was followed. The results showed that, when inoculated with Pst-DC3000, the activity of LOX was increased significantly in RL-treated plants compared with non-treated plants (Fig. 3D). The upregulation of LOX activity exhibited the same tendency as induction of *LOX2*, *LOX3*, and *LOX4* transcripts. These data together demonstrated that LOX was upregulated both in transcription and activity during the defence response induced by RL.

### PIF3 can bind to the sequence of LOX to inhibit its expression, while RL relieves the inhibition

**Fig. 4.**
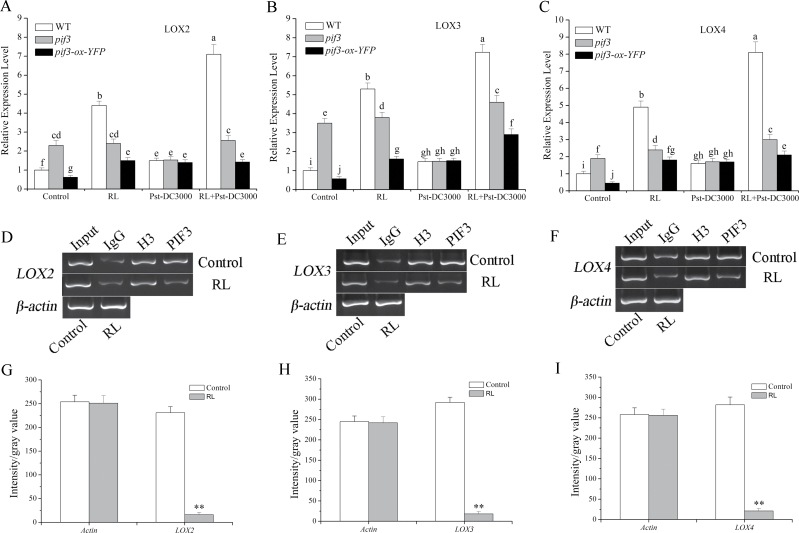
PIF3 inhibits *LOX* transcript expression by binding to its gene sequence. (A–C) PIF3 inhibited *LOX2*, *LOX3*, and *LOX4* transcript levels. Expression levels were detected 4h after different treatments as indicated above using quantitative RT-PCR. *Arabidopsis ACTIN2* was used as an internal control. Expression levels for each treatment were normalized to a RL-treated (0h) plant. (D–F) The binding of PIF3 and LOX gene sequence in normal light (control) or RL-treated (120 μmol photons m^–2^ s^–1^ for 4h) samples of the *pif3-ox-YFP* plant determined by ChIP. The co-immunoprecipitated DNA was detected by agarose gel electrophoresis. Input indicated samples before immunoprecipitation; IgG, H3, and PIF3 indicate samples immunoprecipitated with IgG antibody, H3 antibody and YFP antibody, respectively. (G–I) Quantitative analysis of the *LOX2* (D), *LOX3* (E) and *LOX4* (F) genes of PIF3 samples are shown in (G), (H), and (I), respectively, with Image J software. Three gel photographs were taken for quantitative analysis, and values represent means±SD. Asterisks indicate significant differences between the control and RL treatment (Student’s paired *t*-test: *P<0.05, **P <0.01).

We showed that phyB-mediated *LOX* transcript expression was induced by RL (Fig. 3A–C). PIFs act as negative transcription factors and bind a G-box (CACGTG) DNA sequence element in many light-regulated genes. Hou *et al.*, (2010) demonstrated that the sequence of the *LOX* gene may contain a G-box or similar domain structure, an interesting finding that led us to wonder whether PIFs could directly bind to special sequence in these genes to inhibit their expression. As the most important and widely studied PIF, PIF3 was analysed in our work. As shown in Fig. 3, the *LOX*2, *LOX3*, and *LOX4* transcripts were upregulated under RL and there was a more significant increase following pathogen inoculation. First, we tested expression levels of *LOX*2, *LOX3*, and *LOX4* in *pif3* (T-DNA insertion mutant) and *pif3-ox-YFP* (overexpressing a YFP–PIF3 fusion) plants. The data in Fig. 4A–C, together with that in Fig. 3, illustrated that the function of phyB signalling in the induction of *LOX*2, *LOX3*, and *LOX4* transcripts by RL was achieved through the negative regulation of PIF3. As shown in Fig. 3A–C, all three genes from plants pre-treated with RL showed a more conspicuous transcription level compared with non-pre-treated plants. A ChIP experiment was performed to test whether PIF3 could combine with the DNA sequence of *LOX*2, *LOX3*, and *LOX4*. We performed comparative PIF3 ChIP analyses on *pif3-ox-YFP* lines. ChIPs from the control proved to be highly enriched in *LOX2*, *LOX3*, and *LOX4*, supporting the conclusion that PIF3 was binding to *LOX2*, *LOX3*, and *LOX4* sequences (Fig. 4D–F) and inhibited their expression (Fig. 4A–C). Thus, based on the results, PIF3 can bind to the promoters of *LOX2*, *LOX3*, and *LOX4* to inhibit their expression, and RL relieved this inhibition and promoted their expressions through the phyB–PIF3 signalling pathway.

### MPK3 and MPK6 are responsible for LOX activation during RL-induced defence

**Fig. 5.**
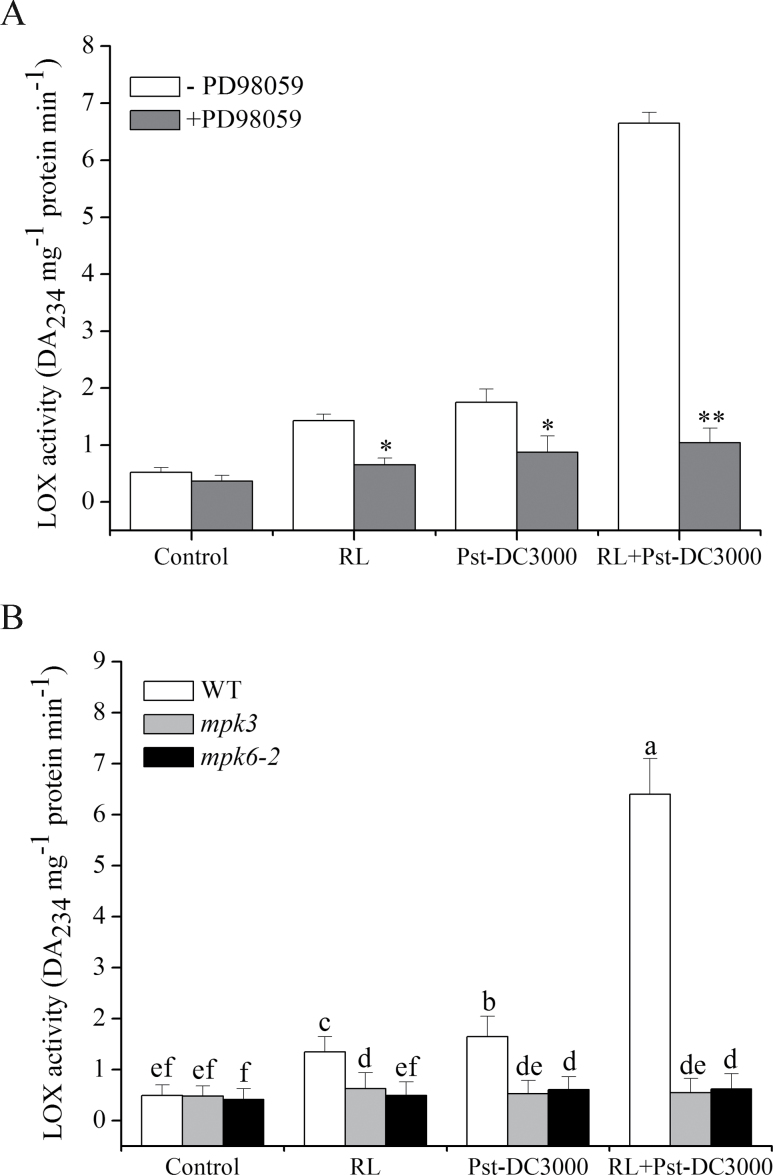
MPK3 and MPK6 are indispensable for LOX activation in *Arabidopsis*. (A) Leaves were pre-incubated with or without PD98059 (20 μM) for 1h, and proteins were then extracted from the leaves at 2h post-treatment. (B) Changes in LOX activity in WT, *mpk3*, and *mpk6-2* plants under different treatments. Plants were treated as follows: control, no treatment; RL, 120 μmol photons m^–2^ s^–1^ for 4h; Pst-DC3000 inoculation, OD_600_=0.01 in 10mM MgCl_2_; RL+Pst-DC3000, inoculation after RL. Different letters indicate statistically significant differences between treatments (Duncan’s multiple range test: *P*<0.05).

In order to investigate whether MAPK cascades were related to the RL-induced activation of *Arabidopsis* LOX, a common inhibitor of the MAPK cascade, PD98059, was used. PD98059 is a selective inhibitor of the MAPK-activating enzyme MEK and consequently of the MAPK cascade (Dudley *et al.*, 1995), which inhibits the activation of MAPK and subsequent phosphorylation of MAPK substrates (Alessi *et al.*, 1995; Kultz *et al.*, 1998; Ye *et al.*, 2013). First, the change in LOX activity was analysed. The results in Fig. 5A indicated that plants pre-irradiated with RL enhanced LOX activity when inoculated with Pst-DC3000, but the increase in LOX activity was effectively inhibited by PD98059. The data indicated that MAPK cascades were involved in LOX activation during the RL-induced defence response, but it remains to be established which specific MPK is involved in this process. MPK3 and MPK6 are reported to be activated in the *Arabidopsis* stress response (Li *et al.*, 2012) and they are both critical in priming plants for full induction of the defence response during induced resistance (Beckers *et al.*, 2009). The LOX activity in leaves of *mpk6-2* (T-DNA insertion mutant) and *mpk3* (MPK3-lacking mutant) plants under different treatments was measured. As shown in Fig. 5B, a substantial increase in LOX activity was observed in RL-treated leaves of WT when inoculated with Pst-DC3000, whereas no significant increase was observed in leaves of *mpk3* and *mpk6-2* plants, suggesting that both MPK3 and MPK6 were indispensable for RL-induced LOX activation when plants were challenged with pathogen.

### Activation of MPK3 and MPK6 is related to Ca^2+^–CaM3

**Fig. 6.**
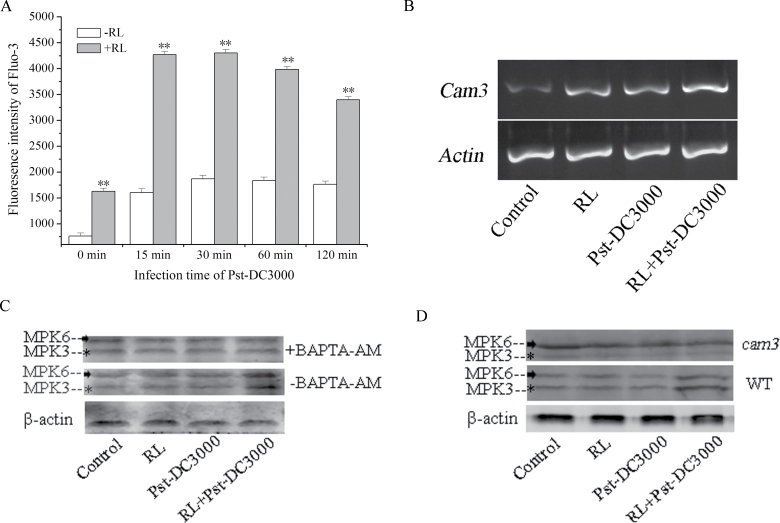
Ca^2+^–CaM3 is involved in activation of MPK3 and MPK6 during the RL-induced defence response. (A) Estimation of RL-induced changes in Ca^2+^ level by flow cytometry using Fluo-3. Protoplasts were incubated with Fluo-3-AM (at a final concentration of 5 μM) for 60min at room temperature and then subjected to flow cytometry analysis. Data represent means±SD of three independent experiments. Statistical analysis was performed with Student’s paired *t*-test. An asterisk indicates a significant difference from the control (**P<0.05). (B) Induction of *CaM3* transcription by RL. Total RNAs were isolated from WT leaves under different treatments and semi-quantitative RT-PCR was performed. (C) MPK3 and MPK6 activity was measured in WT plants with (+BAPTA) or without (–BAPTA) 1mM BAPTA pre-treatment. (D) MPK3 and MPK6 activity was measured in WT plants and *cam3* mutants. Proteins were extracted from leaves treated as follows: control, no treatment; RL, 120 μmol photons m^–2^ s^–1^ for 4h; Pst-DC3000 inoculation, OD_600_=0.01 in 10mM MgCl_2_ for 2h; RL+Pst-DC3000, 2h inoculation after RL. Each data point is the mean±SD of three independent replicates. Different letters indicate statistically significant differences between treatments (Duncan’s multiple range test: *P*<0.05).

The results given above demonstrated that MPK3 and MPK6 were both implicated in RL-induced activation of LOX. Plant MPKs have high homology to mammalian ERK1/2 MPKs, and ERK1/2 antisera that recognize the dually phosphorylated forms (pTEpY) of activated MPKs can be used to monitor plant MPK activity (Li *et al.*, 2012). Hence, the endogenous kinase activity of MPK3 and MPK6 was determined using anti-ACTIVE MAP kinase polyclonal antibody (pTEpY). As shown in Fig. 6C, D, in response to RL, while a transient increase in both the MPK3 activity (43kDa band) and MPK6 activity (47kDa band) were observed in RL-treated WT followed by inoculation, compared with the control, no increase in kinase activity of MPK3 and MPK6 was observed in plants either treated with RL only or inoculated with Pst-DC3000 directly. This indicated that MPK3 and MPK6 were activated during the RL-induced defence response.

We detected an increase in [Ca^2+^]_cyt_ and upregulation of the *CaM3* transcript level (Fig. 6A, B), which may function upstream of activation of MPK3 and MPK6 in response to RL, when plants were inoculated with Pst-DC3000. These observations compelled us to test the effect of application of the Ca^2+^ scavenger BAPTA-AM on activation of MPK3 and MPK6. BAPTA-AM is a lipophilic compound capable of crossing cell membranes; inside the cell, non-specific esterases cleave the AM moiety, thereby forming the ionized Ca^2+^-binding compound BAPTA (Tymianski *et al.*, 1994; Kawano *et al.*, 2000; Yue *et al.*, 2012). The data in Fig. 6C showed that BAPTA-AM inhibited activation of MPK3 and MPK6 effectively, while the activation of MPK3 and MPK6 was impaired in *cam3* (CaM3-lacking mutant) relative to WT (Fig. 6D). Together, MPK3 and MPK6 were apparently activated during RL-induced defence and the signalling pathway was related to Ca^2+^–CaM3.

### LOX is not activated through directly binding with MPK3/MPK6

**Fig. 7.**
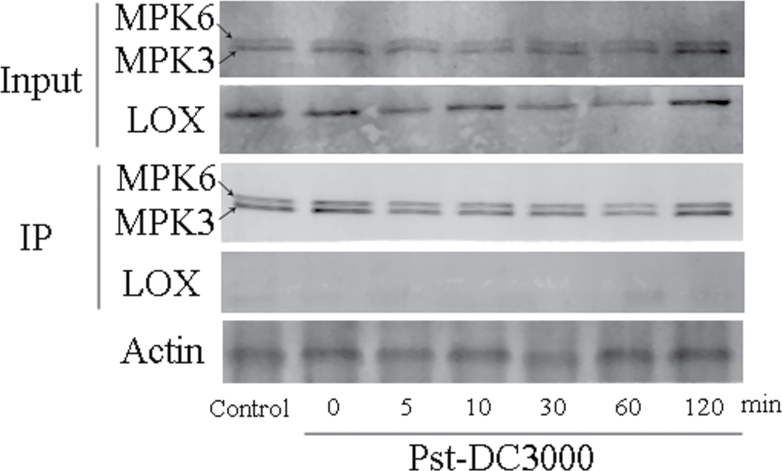
MPK3 and MPK6 are not directly combined with LOX. Co-immunoprecipitation experiments were performed using WT lines. Input indicates samples before immunoprecipitation with anti-ERK antibody, and IP indicates co-immunoprecipitated samples from different treatments. Proteins were extracted from RL-pre-treated leaves at the indicated times after inoculation with Pst-DC3000.

Signalling transduction related to MPKs cascades is generally decoded by phosphorylation of downstream molecules. In our research, induction of LOX activity was concomitant with activation of MPK3 and MPK6, but whether LOX was regulated by MPK3 and MPK6 directly or indirectly was unknown. To gain more information about the regulation pattern, co-immunoprecipitation experiments were performed using WT lines. As shown in Fig. 7, we detected activation of MPK3, MPK6, and LOX before co-immunoprecipitation but no LOX in immunoprecipitates. Therefore, LOX could not be co-precipitated with MPK3 and MPK6, as a direct interaction was not observed in immunoprecipitates.

## Discussion

Szechyńska-Hebda et al. (2010) demonstrated that plants possess a complex and dynamic light training and memory system that involves quantum redox, reactive oxygen species, and hormonal and photo-electrophysiological signalling to optimize light acclimation and defences. In their research, defence responses were induced by excess RL but not blue light. However, the molecular mechanisms underlying the induced process of defences have not been fully resolved. Our findings indicated that phyB plays an important role in the enhancement of defences (Fig. 1). PhyB acts in many aspects of plant growth and development. Recent studies have reported that phyB may participate in the defence response (Griebel and Zeier, 2008; Kazan and Manners, 2011). PhyB usually regulates the expressions of related genes by combining with PIFs. In our study, the data showed that phyB signalling was indispensable for RL-induced activation of LOX (Fig. 3) and PIF3 clearly functioned downstream of phyB (Fig. 4).

LOX is widespread in both animals and plants (Liavonchanka and Feussner, 2006) and catalyses the key step of lipid peroxidation (Gao *et al.*, 2011). It catalyses the production of substances called oxylipins. Oxylipins, a series of versatile molecules, are engaged in many aspects during plant growth and development. The oxylipin synthetic pathway mediates plant defence responses to diverse biotic and abiotic stresses (Blée, 2002; Creelman and Mulpuri, 2002; Howe and Schilmiller, 2002). Oxylipins are believed to play pivotal roles in defences (Krumm *et al.*, 1995; Bate and Rothstein, 1998) and they act as signal molecules and/or protective compounds, or as constituents of cutin (Blée, 2002). Hause *et al.*, (2000) also showed that distinctive oxylipin profiles were produced by different external stimuli and by developmental cues. In this work, we found that RL upregulated LOX both at the transcription level and in terms of activity (Fig. 3), suggesting the possible involvement of phyB-mediated LOX activation in the RL-induced *Arabidopsis* defence response.

PIF3 acts as a negative factor and inhibits expression of some genes by binding to their promoters (Martínez-García *et al.*, 2000; Quail, 2002). ChIP experiments (Fig. 4D–F) indicated that PIF3 binds to gene sequences of *LOX2*, *LOX3*, and *LOX4*, and expression of these three genes was prevented in control plants compared with RL-treated plants. The data above suggested that the gene sequences of *LOX2*, *LOX3*, and *LOX4* may contain a G-box domain or a similar structure. A well-recognized mechanism of phyB signalling pathway is that activated phyB enters into nucleus and combines with PIF3 to target its degradation; this degradation is caused by phosphorylation and ubiquitination of PIF3 (Nicholson *et al.*, 2011; Zhang *et al.*, 2013). In our work, under normal circumstances, PIF3 combined with a region of the *LOX2*, *LOX3*, and *LOX4* gene sequences to suppress their expressions. When plants were exposed to a certain intensity of RL, activated phyB entered into nucleus and caused degradation of PIF3; inhibition *LOX* was relieved, thus leading to an enhancement of expression of these *LOX* genes (Fig. 3A–C).

MPK cascades can be activated by various stimuli and play central roles in the process whereby extracellular stimuli are transduced into intracellular responses (Widmann *et al.*, 1999; Asai *et al.*, 2002; Nakagami *et al.*, 2005). Our experiments demonstrated that MPK cascades also participate in the activation of LOX during the RL-induced defence response (Fig. 5A). Among the various MPK proteins, MPK3 and MPK6 are well-established signalling proteins in *Arabidopsis*, and can be activated by various stimuli. In RL-treated plants, the activation of both MPK3 and MPK6 was proved to be responsible for the upregulation of LOX activity and the subsequent execution of a defence response (Fig. 5B). A typical MPK signalling module consists of three kinases: an MAPKKK, an MAPKK, and an MAPK. MAPKs function at the bottom of the kinase cascade and are activated by MAPKKs through phosphorylation. The activation of MAPKKs is, in turn, regulated by MAPKKKs via phosphorylation. In our study, which MAPKK and MAPKKK are involved in the activation of MPK3 and MPK6 was unclear. MAPKK4/5 is possible engaged upstream of MPK3 and MPK6, because a large body of research has discovered that MAPKK4/5 activates MPK3 and MPK6 during plant pathogen signalling (Meng and Zhang, 2013; Vidhyasekaran, 2014). As an important signal messenger, Ca^2+^ can function upstream of the activation of the MPK cascade under different stimuli (Xing *et al.*, 2008; Wang PC et al., 2010). Under RL, we found increased [Ca^2+^]_cyt_ (Fig. 6A) and upregulation of *CaM3* (Fig. 6B), which functioned in the upstream activation of MPK3 and MPK6 during the defence response. MPKs are proline-directed serine/threonine kinases phosphorylating serine or threonine in the dipeptide motif S/T-P (Bardwell, 2006), *Arabidopsis* LOX sequence show the presence of phosphorylation site for MPK (Taj *et al.*, 2011b), an immunoprecipitation between MPK3/6 and LOX was done. We have not detected the binding of MPK3/6 and LOX at the time point we selected, which may due to the instantaneous interaction between them during defence response (Taj *et al.*, 2011a), which means the time of interaction is too short to capture. Another possible explanation is that MPK3 and MPK6 do not facilitate LOX activation by binding with it directly but may function through downstream WRKY transcription factors. The WRKY transcription factors are also activated by MAPK-dependent phosphorylation and function downstream of MAPK during the defence response (Ishihama and Yoshioka, 2012). Whether WRKY is involved in the activation of LOX and the mechanisms in this regulation pathway needs further research.

Our experimental results showed that when plants pre-irradiated with RL were infected by the pathogen, the activity of LOX was significantly increased compared with that in plants directly inoculated with the pathogen. Whereas a comparative significant increase in *LOX2*, *LOX3*, and *LOX4* was induced in RL-treated plants before pathogen infection, higher levels were induced after infection (Fig. 3). This means that when plants were exposed to RL, transcripts of several *LOX* genes increased significantly, while no obvious changes were detected in activity, indicating that plants are preparing for combat with the pathogen and have become very sensitive to pathogen invasion. Once the pathogen had infected the plant, the plants produced a stronger and more rapid response. As Beckers *et al.* (2009) proposed and demonstrated, accumulation of mRNA was primed for activation of the defence responses. In our study, accumulation of *LOX2*, *LOX3*, and *LOX4* transcripts induced by RL was primed for a later defence response to the pathogen. A stronger LOX activity, subsequent *PR1* expression, and callose deposition were triggered as later defence responses (Figs 2 and 3). Plants primed by treatments that induce resistance show a faster and/or stronger activation of defence responses when subsequently challenged by pathogens or abiotic stresses (Conrath *et al.*, 2002, 2006). As a part of induced resistance responses, priming has been studied in many plants (Kuć, 1987; Zimmerli *et al.*, 2000; Verhagen *et al.*, 2004) for a number of years, but the molecular mechanism of priming has been presented recently (Beckers *et al.*, 2009), and is associated with MPK3 and MPK6 during the development of chemically induced resistance in *Arabidopsis*. In our study, activation of MPK3 and MPK6 promoted LOX activity in the RL-induced defence response.

**Fig. 8.**
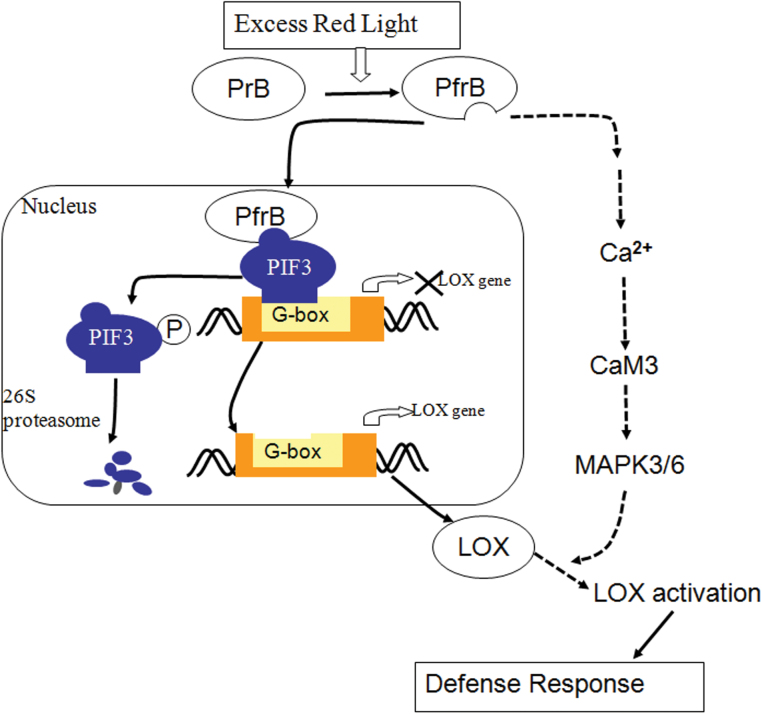
Proposed working model for LOX activation of both transcript levels and activity during the RL-induced defence response. (This figure is available in colour at *JXB* online.)

Our investigations provide evidence that LOX is responsible for defences induced by excess RL. According to the experimental results, a potential cascade of cellular events during enhancement of the defence response is suggested. As summarized in the model presented in Fig. 8, under the condition of RL, phyB is photo-activated and translocates to nucleus; it then binds with PIF3 and promotes its degradation. The degradation of PIF3 promotes expression of *LOX2*, *LOX3*, and *LOX4*, because these genes are inhibited by binding of PIF3 to the *LOX* DNA sequence. When plants are challenged with a pathogen, [Ca^2+^]_cyt_ in cytoplasm is increased rapidly, which activates MPK3 and MPK6, thereby promoting the LOX enzyme capacity. As a result, plants can induce *PR1* expression and callose deposition effectively, which means an enhancement of the defence response. Our results contribute to the corroboration of the signalling mechanism of induced defences by RL and highlight an important role of LOX in the process. Obviously, this method of enhancing the plans defence response is easy to implement and will have wide-ranging use in crop cultivation.

## Supplementary data

Supplementary data are available at *JXB* online.

Supplementary Fig. S1. Impact of different times of RL on *PR1* expression.

Supplementary Fig. S2. Induction of transcription levels of *LOX1*, *LOX5*, and *LOX6* by RL.

Supplementary Fig. S3. Effect of different concentrations of PD98059 on activation of LOX.

Supplementary Fig. S4. Activation of MPK3 and MPK6 during the defence response induced by RL in WT, *mpk6-2*, and *mpk3* plants.

Supplementary Table S1. Primers for several genes.

Supplementary Data

## Abbreviations:

[Ca^2+^]_cyt_: cytosolic calcium concentration
CaM: calmodulin
ChIP: chromatin immunoprecipitation
dpi: days post-inoculation
EL: excess light
ERK: extracellular signal-regulated kinase
LOX: lipoxygenase
MAPK/MPK: mitogen-activated protein kinase
NDGA: 4,′(2, 3-dimethyltetramethylene)dipyrocatechol
phyB: phytochrome B
PIF: phytochrome-interacting factor 3
Pst: *Pseudomonas syringae* pv. *tomato*
RL: red light
RT-PCR: reverse transcription-PCR
SD: standard deviation
WT: wild type.
