# Supplementary material for: Phytochrome B-mediated activation of lipoxygenase modulates an excess red light-induced defence response in Arabidopsis
Source: J Exp Bot. 2014 Jun 10;65(17):4907–18. doi: 10.1093/jxb/eru247 (PMC4144769; doi:10.1093/jxb/eru247)
Supplement: Supplementary Data [file supp_65_17_4907__index.html]

Phytochrome B-mediated activation of lipoxygenase modulates an excess red light-induced defence response in Arabidopsis — Phytochrome B-mediated activation of lipoxygenase modulates an excess red light-induced defence response in Arabidopsis — Supplementary Data 

# Phytochrome B-mediated activation of lipoxygenase modulates an excess red light-induced defence response in *Arabidopsis*

## Supplementary Data

Data files

**Files in this Data Supplement:**

- Supplementary Data - Supplementary Data
