# Supplementary material for: Phytochrome B-mediated activation of lipoxygenase modulates an excess red light-induced defence response in Arabidopsis
Source: J Exp Bot. 2014 Jun 10;65(17):4907–18. doi: 10.1093/jxb/eru247 (PMC4144769; doi:10.1093/jxb/eru247)
Supplement: Supplementary Data [file supp_eru247_jexbot123117_file001.pdf]

Journal of Experimental Botany

Phytochrome B-mediated Activation of Lipoxygenase Modulates Excess red light-induced Defense Response in Arabidopsis

Yuanyuan Zhao, Jun Zhou, Da Xing*

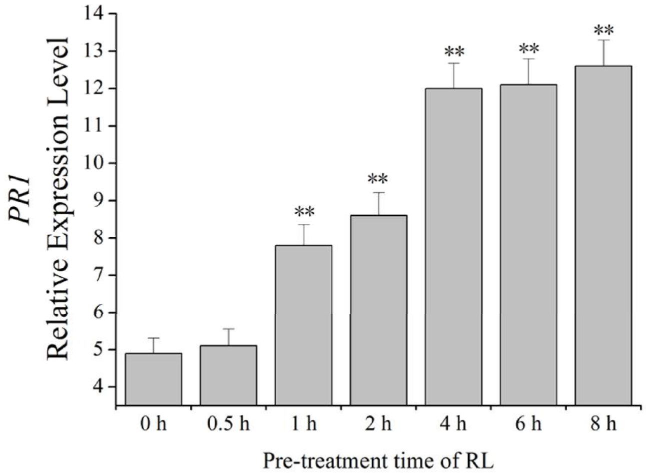

Figure S1

Fig. S1. Impact of different time of RL on *PR1* expression. Quantitative RT-PCR data show the expression of the *PR1* gene in WT *Arabidopsis*. Total RNA was extracted from leaves 4 h post inoculation by Pst-DC3000, plants were pre-treated with different time of RL as indicated. *Arabidopsis ACTIN2* was used as an internal control. Expression levels for each treatment were normalized to a RL-treated (0 h) plant. Values represent means ±SD of three independent experiments. Asterisks (*) indicate a significant difference from the (0 h) at * P < 0.05 or ** P < 0.01 by *t*-test.

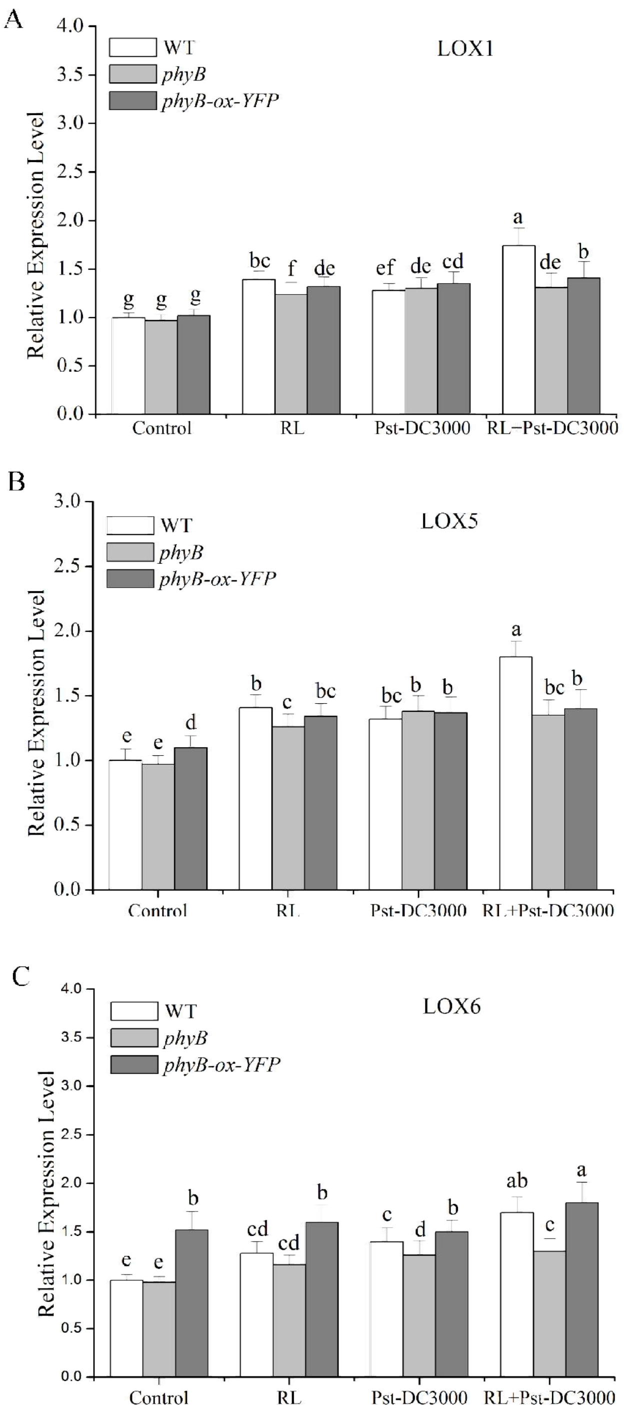

Figure S2

Fig. S2. Induction of transcription levels of *LOX1, LOX5, LOX6* by RL. (A)-(C) Transcript levels of *LOX1, LOX5, LOX6* in wild-type, *phyB*, *phyB-ox-YFP* plants. Total RNA were extracted from the leaves of full-grown *Arabidopsis* after different treatments as follows: control (no treatment), RL (120 μmol photons $m^{-2}s^{-1}$; 4 h), Pst-DC3000 inoculation (OD_{600}=0.01 in 10 mM $MgCl_2$), RL+Pst-DC3000 (inoculation after RL). *Arabidopsis ACTIN2* was used as an internal control. Different letters indicate statistically significant

differences between treatments (Duncan's multiple range test: $P < 0.05$). Values represent means ±SD of three independent experiments.

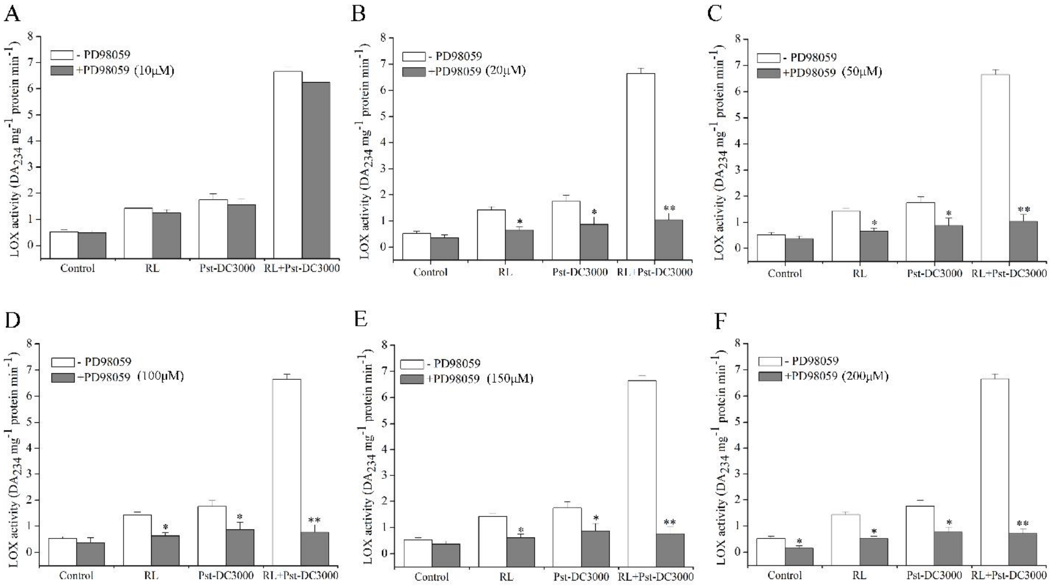

Figure S3

Fig. 3. Effect of different concentrations of PD98059 on activation of LOX. Leaves were pre-incubated with or without PD98059 for 1 h, and then proteins were extracted from the leaves 2 h post treatment. Concentrations of PD98059 in (A)-(F) were 10 μM, 20 μM, 50 μM, 100 μM, 150 μM and 200 μM.

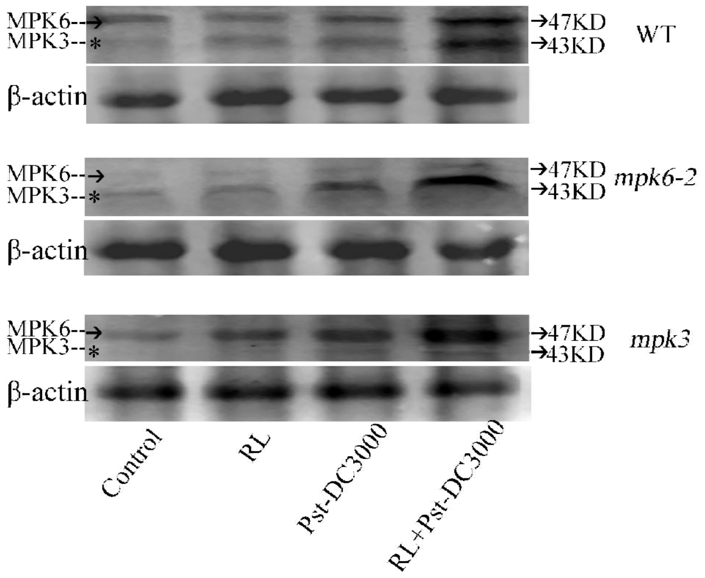

Figure S4

Fig. S4. Activation of MPK3 and MPK6 during defense response induced by RL in the WT, *mpk6-2* and *mpk3* plants. Proteins were extracted from the leaves treated as follows: control (no treatment), RL (120 μmol photons $m^{-2}s^{-1}$; 4 h), Pst-DC3000 inoculation (OD_{600}=0.01 in 10 mM $MgCl_2$, 2 h), RL+Pst-DC3000 (2 h inoculation after RL). The experiment was repeated three times with similar results.

Table S1. Primers for several genes.

Gene name	Primer
LOX1	U:GATAGTGAATCATACGGTAACCAGA<br>D:AGGATGAGGCAAGCTCAGCTCTAT
LOX2	U:CACCTGACGAAGAGTACATTGG<br>D:GTAACACCATGCTCAGAGGTAGG
LOX3	U:TGAACATTGAGAGAGTCAAGACTTTT<br>D:AGATAGTTCGAGTAGCATAGGCTTTGC
LOX4	U:TCGCTAACTTTGGTGAGATCGATAG<br>D:TCGTCATCTCGAAGCATGCATATT
LOX5	U:CATACGAACAGAGCACATAGAATCA<br>D:AAACTTTGCTGACCGATCCATATGA
LOX6	U:GCTTGAAATCAATGCTCGTGCG<br>D:GTCAATCACAAGCCTCACGCCAC
CaM3	U:GCTCTAGAACAGGTTTCACGAAAAGGAGA<br>D:CGGGATCCCTTAGCCATCATGACCTTAAC
Actin2	U:AGAGATTCAGATGCCCAGAAGTCTTGTTCC<br>D:AACGATTCCTGGACCTGCCTCATCATACTC
PR1	U:CATACACTCTGGTGGGCCTT<br>D:GACCACAAACTCCATTGCAC
